# How Gas Is Made

**Published:** 1874-06

**Authors:** 


### HOW GAS IS MADE.

It is very easy to make gas, but it costs much trouble to purify it, that it may burn well and give off no noxious odors. Below is a sort of gas catechism which conveys a good deal of important "light" on the subject.

"How do they make gas?"

"First, they put about two bushels of bituminous coal in a long air tight retort. This retort is heated red-hot, when the gas bursts out of it, as you see it burst out of soft coal when on the parlor fire. The gas passes off through pipes. A ton of coal will make ten thousand cubic feet of gas. The gas, as it leaves the coal, is very impure."

"How do they purify it?"

"First, while hot, it is run off into another building; then it is forced through long perpendicular pipes, surrounded with cold water. This cools the gas, when a good deal of tar condenses from it and runs down to the bottom of the perpendicular boiler half full of wood laid crosswise. Then ten thousand streams of cold water are spurted through the boiler. Through the mist and rain, and between the wet sticks of wood the gas passes, coming out washed and cleansed. The ammonia condenses, joins the water and falls to the bottom."

"What next?"

"What next—the gas is purified. It is passed through vats of lime and oxide of iron, which takes out the carbonic acid and ammonia."

"What next?"

"The gas is now pure. It passes through the big station meter, then through the main and pipes till it reaches the gas jets in your room. Then it burns, while you all scold because it does not burn better."

